# Horizontal acquisition and a broad biodistribution typify simian foamy virus infection in a cohort of *Macaca fascicularis*

**DOI:** 10.1186/1743-422X-10-326

**Published:** 2013-11-01

**Authors:** Simon Hood, Jane L Mitchell, Meera Sethi, Neil M Almond, Keith L Cutler, Nicola J Rose

**Affiliations:** 1 Division of Virology, National Institute for Biological Standards and Control, Blanche Lane, Medicines and Healthcare products Regulatory Agency, South Mimms, Potters Bar, Hertfordshire EN6 3QG, UK

## Abstract

**Background:**

Foamy viruses are non-pathogenic *in vivo* and naturally infect all species of non-human primates (NHP). Simian foamy viruses (SFV) are highly prevalent in both free ranging and captive NHP but few longitudinal studies have been performed to assess the prevalence and biodistribution of SFV within captive NHP.

**Method:**

LTR and pol gene along with Gag antibody detection were undertaken to identify infection in a cohort of over 80 captive macaques.

**Results:**

The prevalence of SFV was between 64% and 94% in different groups. Access to 23 dam-infant pairs allowed us to reveal horizontal transfer as the dominant route of SFV transmission in our cohort. Further, analysis of SFV from a range of tissues and blood revealed that macaques as young as six months old can be infected and that proviral biodistribution increases with age.

**Conclusions:**

These are the first data of this type for a captive cohort of cynomolgus macaques.

## Introduction

Foamy viruses, or spumaviruses, are the only genus within the *Spumaretrovirinae* sub-family of the *Retroviridae*[1]. Foamy viruses have been isolated from non-primate species and various New and Old World primate species (reviewed in [1]–[6]). The simian foamy viruses are hypothesised to have co-speciated with non-human primates (NHP) over an estimated 60 million years enabling them to become well adapted to their hosts [1,4,7,8]. Each natural host harbours its own unique monophyletic strains of the virus [1,9].

SFV is highly prevalent in both captive and free-ranging NHP populations [10]–[12] and infection is life-long. Transmission of SFV between NHP species [13], and zoonotic infections from NHP species to humans have been documented with no overt pathological effect, though virus-specific antibodies are raised [2,14]–[19]. As yet human-to-human transmission of SFV has not been reported [15,18]. A number of studies have shown SFV to be highly prevalent in a range of captive NHP which provides a good opportunity to study virus transmission [7,10,14,16,20]. The transmission of SFV between NHP has been thought to primarily occur from transfer of saliva through biting or licking (reviewed in [1,11,21]–[23]). One study in a wild Western chimpanzee population, with follow-up investigations in a sub-set of the animals, demonstrated a high degree of vertical SFV transmission and suggests this accounts for many primary infections [24]. There has been one reported longitudinal study of SFV transmission in captive NHP colonies in which SFV seroprevalence was assessed in a population of Tonkean macaques (*Macaca tonkeana*; [10]). Within the population 4.7% of immature macaques, 43.7% of subadult macaques and 89.5% of adult macaques were infected. Most cases of seroconversion occurred after seven years of age and were thought to be as a result of transmission via severe bite wounds. Six different strains of SFV were detected in the Tonkean macaque colony and within any specific animal no *in vivo* viral evolution was observed. Transmission data for SFV in captive cynomolgus macaques is currently not available.

Foamy viruses are non-pathogenic *in vivo*, however, there is little in the literature regarding the overall biological burden of SFV, though replicating virus has been detected in the oral mucosa of healthy individuals, and the jejunum and the mesenteric lymph nodes in immunosuppressed hosts [25]. SFV-specific antibodies and viral RNA isolated from chimpanzee faeces suggest that gut epithelia may be a further site of SFV replication [12]. By contrast, SFV proviral DNA can be isolated from most tissue types including blood [11,25,26]. It is possible that reservoirs of non-replicating virus in a host could reactivate to become active, transmissible virus, allowing spread within a colony and the generation of different sequence variants.

The cynomolgus macaque (*M*. *fascicularis*) is used widely in biomedical research. In particular, we and others have used this species as a model for HIV infection given their susceptibility to simian immunodeficiency virus (SIV). We have previously created a cohort of young cynomolgus macaques from which simian retrovirus, type 2 (SRV-2) and simian T-lymphotropic virus, type I (STLV-I) have been eliminated primarily by PCR based selection [27]. These new colonies of young macaques offered us an excellent opportunity to study SFV infection in isolation from SRV-2 and STLV-I. Critically, the availability of dam-offspring pairs allowed the evaluation of the occurrence of vertical acquisition of SFV against horizontal acquisition, which has not been reported for cynomolgus macaques previously. We performed a study of proviral load and distribution to characterise the extent of possible virus reservoirs in our macaques. Sequence variation in the *pol* gene was used to assess likely routes of virus transmission within this captive cohort. We determined that animals as young as six months in our cohort could be seropositive and harbour provirus. In three groups of macaques, we showed by sequence analysis that transmission of SFV variants was primarily horizontal. SFV infection, as measured by molecular and serological approaches, correlated positively with increasing age. Proviral DNA was isolated from a range of tissues and while the proviral load in individual tissues did not correlate with age, older animals had a broader biodistribution of SFV.

## Results

### SFV prevalence in a group of age-stratified cynomolgus macaques (Study Group 1; SG1)

To determine the prevalence of SFV in a cross-section of macaques of different ages, a group of 25 macaques was stratified according to age. The presence of SFV provirus in PBMC-derived DNA was determined by PCR amplification of 5′ LTR sequence (this is largely invariant and thus gives a high probability of virus detection) and a region of the *pol* gene. Specific anti-SFV antibodies were detected by ELISA. Of the 25 macaques, 21 were identified as harbouring SFV provirus by PCR and anti-SFV antibodies by ELISA. The data from the three assays correlated for all but a single macaque (492) from which SFV LTR sequences were not amplified. The sole infant, and all of the middle age and older adult macaques, were SFV positive. Of the eight young adult macaques (four female, four male), two males were SFV negative; of the four male juvenile macaques, two were negative (Table 1), suggesting either a low viral load below the detection limits of the assay or a true lack of infection to date in these animals.

**Table 1:** SFV status of the 25 cynomolgus macaques in Study Group 1

**Macaque identification**	**Age/years**	**Age classification**	**Gender**	**SFV status**		
				**PCR:Pol**	**PCR:LTR**	**ELISA**
492	27.5	Old adult	M	+	-	+
838	23.7		M	+	+	+
055D	22.0		M	+	+	+
023G	20.5		F	+	+	+
768B	20.3		F	+	+	+
022G	20.2		F	+	+	+
017K	16.8	Mid adult	M	+	+	+
691C	16.4		F	+	+	+
786C	16.3		F	+	+	+
005L	16.1		M	+	+	+
768E	15.5		F	+	+	+
012M	14.9		M	+	+	+
936DBE	10.0	Young adult	F	+	+	+
942	8.0		F	+	+	+
905	8.0		F	+	+	+
M980	7.9		M	-	-	-
I458A	7.6		M	+	+	+
M304	7.0		M	+	+	+
003L	7.0		F	+	+	+
990	6.0		M	-	-	-
956ED	2.1	Juvenile	M	+	+	+
983BB	2.0		M	-	-	-
958EH	1.7		M	-	-	-
N4G	1.4		M	+	+	+
980ABAF	0.8	Infant	F	+	+	+

### Biological burden of SFV (Study Group 2; SG2)

**Figure 1:**
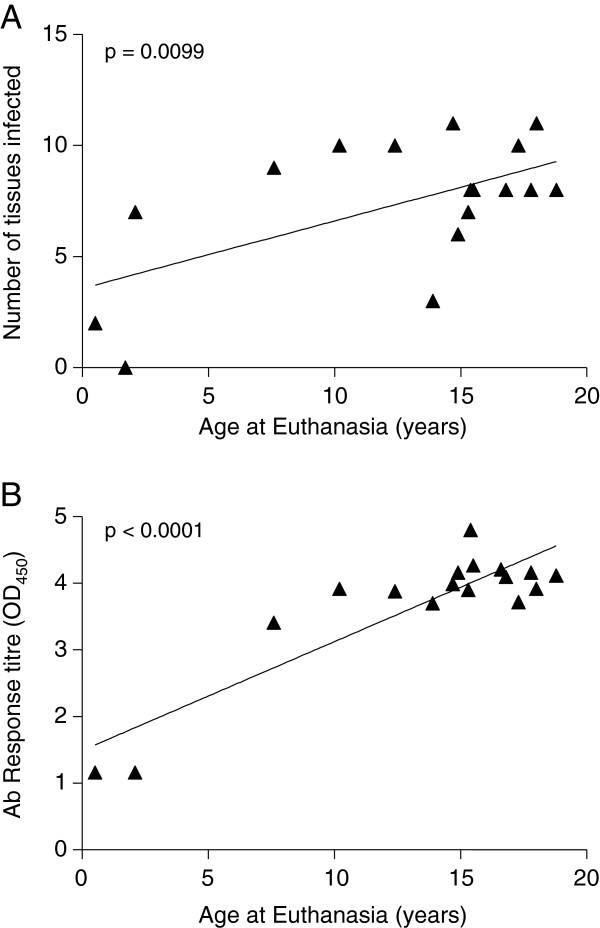
Correlation between (A) the number of tissues positive for SFV provirus and age and (B) the antibody titre and age.

To determine the range of tissues infected in each age group and thus predict which tissues may be infected early following challenge, the presence of SFV *pol* sequences was determined in 11 tissues from 3 juveniles and 15 adult macaques (age range 7 – 20 years), termed Study Group 2; SG2. A one-year old infant (958EH) was negative for SFV in all tissues. The remaining two infants were SFV positive. SFV sequences were detected in the mesenteric lymph node (MLN) and the salivary gland lymph node (SGLN) of a six-month old macaque (M955C). A two-year old macaque (956ED) also had detectable SFV sequences in the MLN and SGLN and provirus was also found in the kidney, liver, small and large intestine and the salivary gland. SFV provirus was detected in seven or more of the eleven tissues tested from all of the adult macaques with viral sequences identified most frequently in the spleen and liver and two of the macaques had SFV infection in all 11 tissues (Table 2). While there was no overt correlation between cumulative viral burden or individual tissue viral burden and age of animal, as age increased a greater number of tissues was found to harbour viral sequences (Figure 1A; p = 0.0099), possibly as a consequence of changes in an older animal’s immune response leading to a reduced ability to control the colonisation of an organ by the virus. Furthermore, animals were more likely to be seropositive (Figure 1B; p <0.0001).

**Table 2:** Tissues infected by SFV

			**Proviral load in tissues (copies/15000 cells)**										
**Macaque**	**Age (years)**	**Total**	**Kidney**	**Tongue**	**PLN**	**MLN**	**Lung**	**Liver**	**Salivary gland**	**Spleen**	**Large intestine**	**Small intestine**	**SGLN**
M955C	0.5	1.00				0.50							0.50
958EH	1												
956ED	2	6.21	0.50			0.50		3.21	0.50		0.50	0.50	0.50
M126	7	14.60	0.50	3.60		2.42	1.00	0.50	0.50	2.00	0.50	3.59	
963DBE	10	263.11	2.94	0.50	18.56	34.29	3.82	28.60	5.24	12.45	130.39		26.33
979B	12	150.94	10.56	21.59	1.13	2.27	6.59	4.99	0.50	6.51	37.47	59.32	
176ABA	13	56.83		3.98		49.86				3.00			
024L	14	6.47	3.52		0.50	0.38		0.50	0.50	1.08			
604F	14	118.90	0.50	58.36	13.08	7.20	8.19	0.39	1.16	3.82	14.27	11.45	0.50
088AD	15	39.05	2.31			1.96		9.65	1.25	6.80	2.57	14.51	
604E	15	26.45	0.63		0.50	1.19	1.02	12.31		6.89	1.16	2.75	
768E	15	44.68	8.66				11.84	1.63	0.29	9.91	11.65	0.21	0.50
017K	16	24.92	1.84	4.49			0.50	1.00	1.13	4.90	10.56		0.50
184E	17	84.23	5.06	41.86	2.24		1.28	3.64	0.57	3.19		26.39	
654D	17	150.63	6.31	55.18	55.79		10.64	2.95	2.74		9.42	7.60	
820A	18	58.78	0.53	45.25		0.50	0.50	0.50	0.85	0.50	9.04	0.50	0.61
085E	18	366.32	7.11	38.87	14.06	4.67	6.16	6.55	2.86	2.37	24.80	258.36	0.50
768B*	20	ND	+			+	+	+	+	+		+	+

The tissues identified as SFV infected by Pol PCR in cynomolgus macaques are presented. *PLN*, peripheral lymph node; *MLN*, mesenteric lymph node; *SGLN*, salivary gland-associated lymph node; *, quantified proviral loads not available for 768B; *ND*, not determined.

### Prevalence of SFV in cohorts of young macaques (Study Group 3; SG3)

To assess the prevalence and transmission of SFV in young macaques, three cohorts (CA5, CA7, CA8) comprising individuals between one and three-years of age at time of colony formation, were studied. Macaques were screened for SFV provirus prior to, and on the day of amalgamation, and on up to four occasions over a 45-month period thereafter.

In CA5 seven founder animals tested positive for SFV prior to amalgamation (Table 3). Eleven macaques became infected over the 33-month screening period. There were 13 births in the colony. An SFV-positive dam did not necessarily give birth to infected offspring, e.g. one SFV-positive multiparous dam (768EI) was associated with two SFV-negative and one SFV-positive offspring (all single births). In CA7, four of the nineteen juvenile founder animals were SFV positive prior amalgamation (Table 4). By the end of the 45-month study 14 previously negative macaques were positive for SFV provirus and had seroconverted, including a previously negative animal transferred from CA5 (109HCE). The majority of new SFV infections occurred after month 33. In CA8 (Table 5), five founder animals were SFV positive. An additional male (980ABAE, SFV-positive) was introduced to the colony 11 months post-formation. Two dams tested positive at a time point that was coincident with their first pregnancy at month 24. Five of eight SFV-positive dams had offspring that tested negative for virus at the first time point. One dam and offspring pair tested negative at the time of earliest post-partum sampling. A further nine breeding females became infected by the end of the 45-month study, suggesting that detectable SFV can coincide with sexual maturity. Accounting for births and removal of animals, the SFV prevalence at the final time point was 64% in CA5, 94% in CA7 and 86% in CA8.

**Table 3:** SRV status of macaques in colony CA5

**Colony**	**Macaque**	**Gender**	**SFV status**^**1**^				
			**-34**	**0**	**13**	**20**	**33**
CA5	954FE	M	-	-	+	+	+
	109HCE^‡^	M	-	-			
	449IE	M	+	+			
	028GCE	F	+	+	+	+	+
	979BAC	F	+	+	+	+	+
	049JI	F	+	+	+	+	+
	357GH	F	+	+	+	+	+
	040JK	F	+	+			
	548FBF	F	-	-	+	+	+
	680BBG	F	-	-	-	-	+
	868CF	F	-	-			
	768EI*	F	-	-	+	+	+
	768EIA^†^	M			+	+	
	768EIB^†^	F				-	
	768EIC^†^	F					-
	019GG*	F	-	-	-	-	+
	019GGB^†^	M				-	-
	019GGC^†^	M					-
	406AO*	F	-	-	+	+	+
	406AOA^†^	M				+	+
	406AOB^†^	M					-
	633AO*	F	-	+	+	+	+
	633AOA^†^	F				-	
	633AOB^†^	F					-
	969GI*	F	-	-	-	-	+
	969GIB^†^	F					-
	053GF*	F	-	-	-	-	+
	053GFB^†^	F					-
	109HM*	F	-	-	-	-	+
	109HMC^†^	F					-
	980BAC*	F	+	+	+	+	+
	980BACB^†^	F					-

SFV status of macaques is shown at different periods (^1^months) from time of colony creation. Status was determined from PCR and ELISA data. *, dam and ^†^offspring groupings. No given data indicates either a period prior to the birth of an infant (^†^) or prior to addition/following removal of a breeding macaque. ^‡^Macaque 109HCE was transferred between colonies CA5 and CA7.

**Table 4:** SRV status of macaques in colony CA7

**Colony**	**Macaque**	**Gender**	**SFV status**^**1**^					
			**-1**	**0**	**11**	**24**	**33**	**45**
CA7	040JL	M	+	+	+	+	+	+
	633AP	M	-	-	-	+	+	+
	109HCE^‡^	M					+	
	398BAK	F	+	+	+	+	+	+
	203EM	F	+	+	+	+		
	164LI	F	+	+	+	+		
	605CF	F	-	-	+	+	+	+
	395BBF	F	-	-	-	+	+	+
	518FF	F	-	-	-	-	+	+
	969GK	F	-	-	-	-	-	+
	980BAE	F	-	-	-	-	-	+
	817DH	F	-	-	-	-	-	+
	691CAF	F	-	-	-	-	-	+
	357CCI	F	-	-	-	-	-	+
	545ACE	F	-	-	-	-	-	-
	055HG	F	-	-	-	-	-	+
	398BBH	F	-	-	-	-	-	+
	357GI	F	-	-	-	-	-	+
	456DBH	F	-	-	-			
	802HAH*	F	-	-	-	-	-	+
	802HAHA^†^	M						+

SFV status of macaques is shown at different periods (^1^months) from time of colony creation. Status was determined from PCR and ELISA data. *, dam and ^†^offspring groupings. No given data indicates either a period prior to the birth of an infant (^†^) or prior to addition/following removal of a breeding macaque. ^‡^Macaque 109HCE was transferred between colonies CA5 and CA7.

**Table 5:** SRV status of macaques in colony CA8

**Colony**	**Macaque**	**Gender**	**SFV status**^**1**^					
			**-1**	**0**	**11**	**24**	**33**	**45**
CA8	980ABAE	M			+	+	+	+
	N31D	F	+	+	+	+	+	
	028LG	F	+	+	+	+	+	+
	980ABAF	F	+	+	+	+	+	+
	436FJ	F	+	+	+			
	768BN	F	-	-	-	-	+	+
	036KH	F	-	-	-	-	-	+
	542BG	F	-	-	-	-	-	+
	458HI	F	-	-				
	N31G^†^	M					-	
	962DF*	F	+	+	+	+	+	+
	962DFA^†^	F						-
	658HF*	F	-	-	-	+	+	+
	658HFA^†^	M					-	
	004KG*	F	-	-	-	+	+	+
	004KGA^†^	M					-	
	034KK*	F	-	-	-	-	-	+
	034KKA^†^	F					-	
	548FBG*	F	-	-	-	-	-	+
	548FBGA^†^	M						-
	109HCF*	F	-	-	-	-	-	+
	109HCFA^†^	M						-
	868CG*	F	-	-	-	-	-	+
	868CGA^†^	F						+
	037KJ*	F	-	-	-	-	+	+
	037KJA^†^	M						+
	978AJ*	F	-	-	-	-	+	+
	978AJA^†^	F						+

SFV status of macaques is shown at different periods (^1^months) from time of colony creation. Status was determined from PCR and ELISA data. *, dam and ^†^offspring groupings. No given data indicates either a period prior to the birth of an infant (^†^) or prior to addition/following removal of a breeding macaque.

### SFV sequence distribution

**Figure 2:**
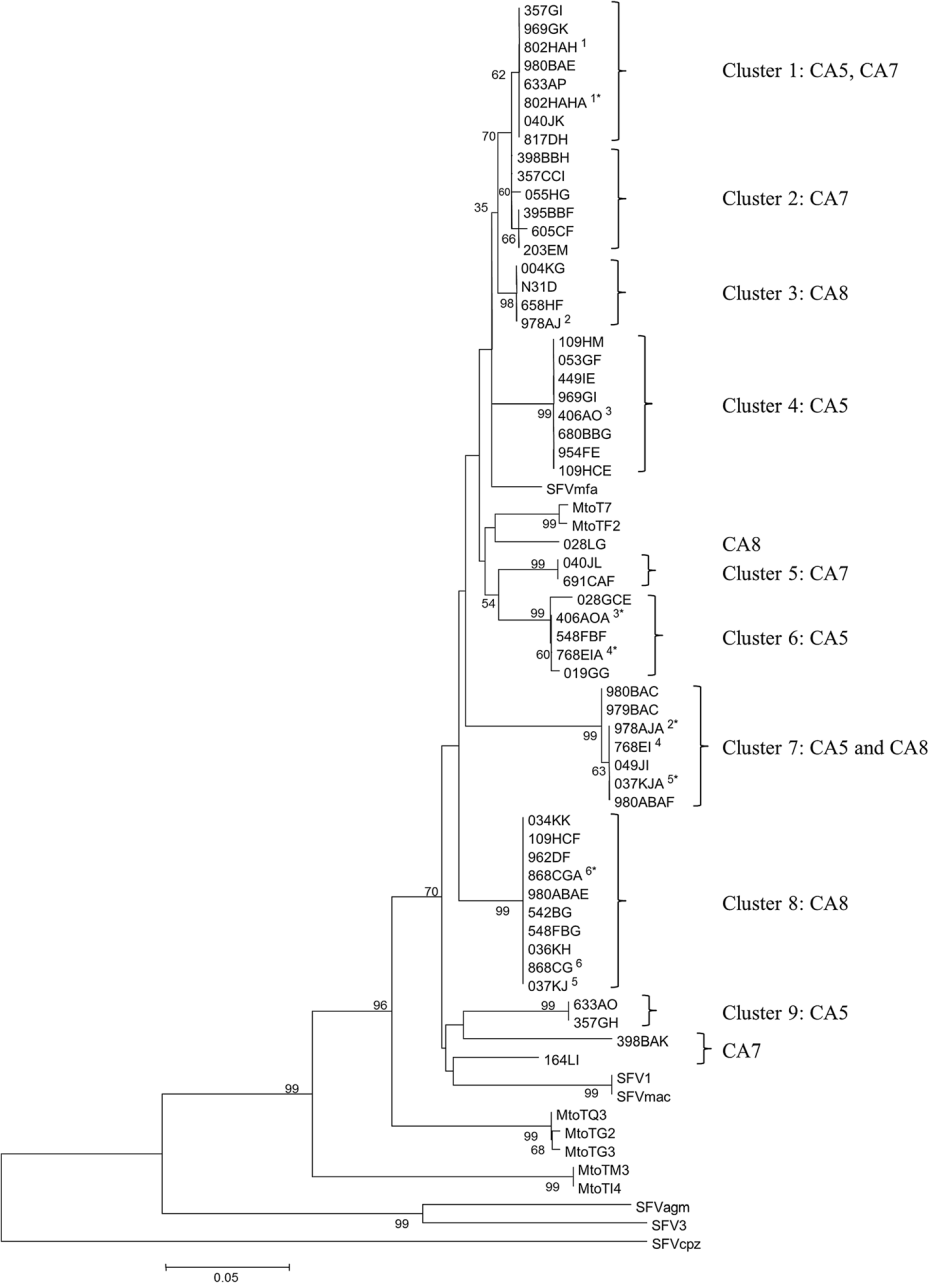
**Evolutionary relationships between the 464-bp SFV *pol *sequences for the cohort of cynomolgus macaques.** The evolutionary distances were calculated using the Neighbor-Joining method and are shown as the number of base substitutions per site. The optimal tree is shown. The percentage of replicates in which the sequences clustered together in the bootstrap test (1000 replicates) is shown next to the branches. Branches corresponding to partitions reproduced in less than 50% bootstrap replicates are collapsed. The tree was rooted using a SFVcpz sequence from Genbank, accession number, U04327. SFV sequences from rhesus macaque (SFVmac, accession number X83292; SFV1, X54482), cynomolgus macaque (SFVmfa, AY686197), Tonkean macaques (SFVMtoT7, DQ354076; SFVMtoTM3, DQ354079; SFVMtoTQ3, DQ354073), chimpanzee and African green monkey (SFVagm, X8329; SFV3, M74895) were included for reference. The main nine clusters are indicated and the groups from which the animals are derived are indicated. Dam-infant pairs used to illustrate the reduced emphasis of vertical transmission of SFV in the study (see text) are indicated by a superscript Roman numeral for the dam, and the corresponding numeral with an asterisk for the infant.

To investigate likely transmission routes of SFV in these colonies, we studied the distribution of SFV sequence variants in infected macaques using DNA samples retrieved from blood at the first time point infection was noted. The *pol* gene was selected as this is known to show sequence variation in populations, as opposed to the largely invariant LTR sequences. A 464-bp region of the SFV *pol* gene from 55 infected macaques from colonies CA5, CA7 and CA8 was bulk sequenced yielding a total of 20 sequence variants. Phylogenetic analysis of the SFV sequences alongside reference sequences available from various species (rhesus, cynomolgus and tonkean macaques; chimpanzee; African green monkey) indicated that all sequences generated clustered with the Asian macaque SFV isolates, as anticipated. The SFV sequences from the colony macaques fall into nine main clusters (Figure 2) with a further three individual SFV sequences (from macaques 164LI, 398BAK (both CA7) and 028LG (CA8)) falling outside of these groups. The clusters primarily featured sequences specific to a colony. Two exceptions were the sequence isolated from macaque 040JK in colony CA5 which clustered with a group of sequences identified in macaques from CA7 and the sequence variant from macaques 980ABAE, 037KJA and 978AJA in colony CA8 which clustered with sequences isolated from the CA5 colony.

These data strongly suggest that SFV was transmitted from both males and females. Of the six SFV-positive offspring born into the three colonies only two (802HAHA and 868CGA) shared a viral sequence with their dam. In each case the same sequence was identified in one of the potential sires. The remaining four SFV positive offspring (037KJA, 406AOA, 768EIA and 978AJA) had sequences different from those infecting their dams and the potential sire (Figure 2). An understanding of the nature and distribution of sequences in the colonies, including the dam-offspring pairs, highlights that transmission between animals is more likely than vertical transmission.

**Figure 3:**
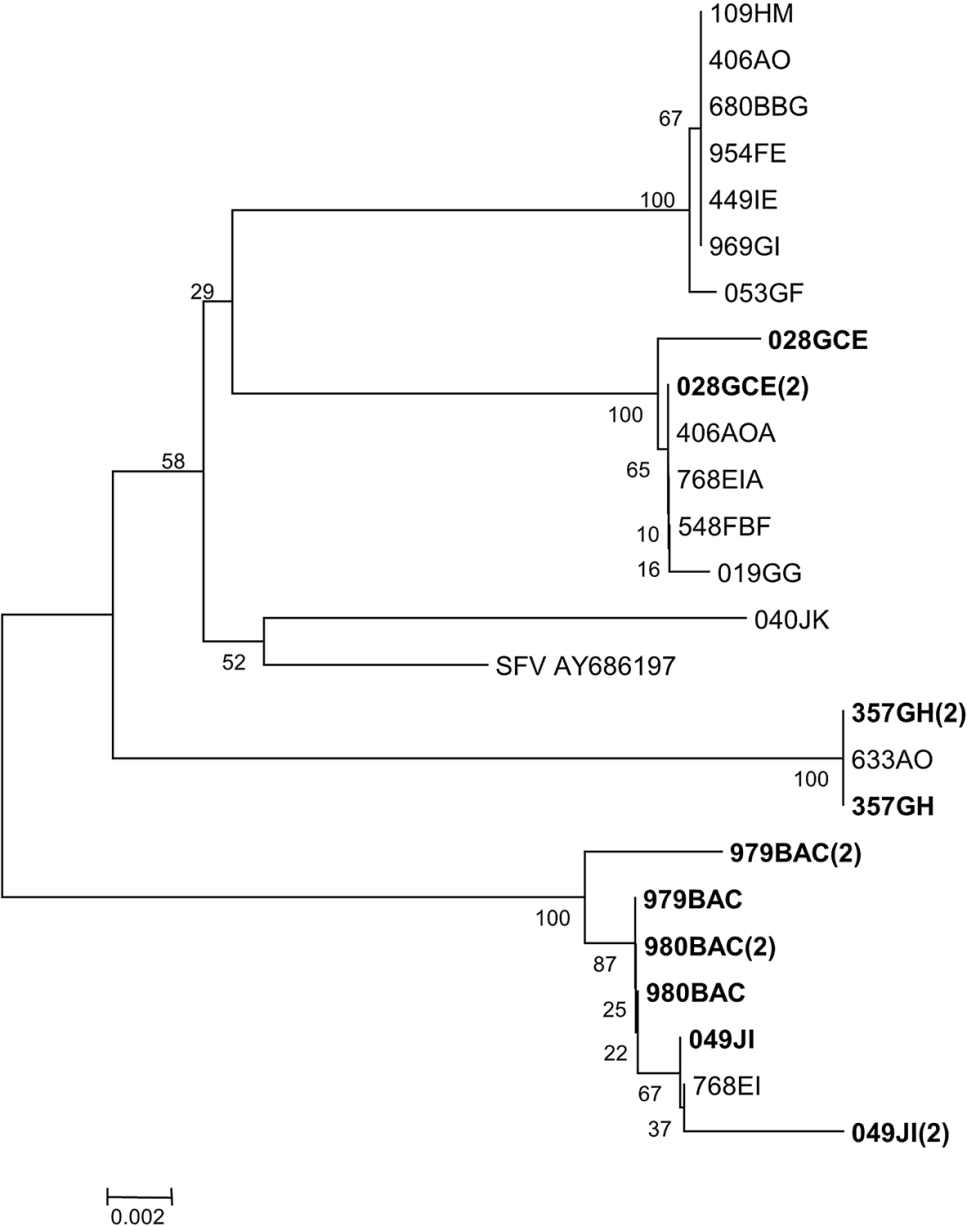
**SFV *pol *sequences in five macaques over time.** The relationship between SFV *pol* sequences is shown for CA5 macaques to illustrate changes between two time points in five macaques (shown in bold). The evolutionary distances were calculated using the Neighbor-Joining method and are shown as the number of base substitutions per site. The percentage of replicates in which the sequences clustered together in the bootstrap test (10000 replicates) is shown next to the branches. Branches corresponding to partitions reproduced in less than 50% bootstrap replicates are collapsed. The tree is drawn to scale, with branch lengths in the same units as those of the evolutionary distances used to infer the phylogenetic tree. The evolutionary distances were computed using the Maximum Composite Likelihood method and are in the units of the number of base substitutions per site.

Five CA5 group macaques (980BAC, 357GH, 028GCE, 979BAC and 049JI) had had tested positive at five time points spanning a 67-month period (Table 3). Bulk sequence data obtained from the first and last time points were compared for each animal alongside the variants identified in the remaining 14 infected animals of the colony. At the amino acid level, eight variants (A-H; GenBank accession numbers JX126870–JX126877) were identified in this Pol fragment sequence (Table 6) revealing 16 polymorphic sites. The number of macaques harbouring these sequences varied (A=6; B=3; C=1, D=1, E=1, F=1; G=2; H=4). Two positions in which all sequences differed from the reference strain (A821T, S865A) suggest that these occurred in the parental SFV strain and may have become fixed in our cohort of macaques. On analysis of the second time point for the sub-group of five long-term infected macaques, three further variant sites were identified. Evolutionary analysis confirmed that the CA5 group macaque sequences broadly clustered into four groups (Figure 3) as suggested by analysis of the whole colony (Figure 2).

**Table 6:** SFV Pol amino acid sequences for five animals with long-term infection from colony CA5

			**Amino acid position of SFV (Pol)**																		
		Time Point*	**781**	**788**	**790**	**795**	**797**	**799**	**801**	**802**	**813**	**821**	**829**	**847**	**853**	**856**	**857**	**865**	**866**	**870**	**902**
	**SFVmfa**		**N**	**N**	**K**	**D**	**K**	**M**	**E**	**R**	**A**	**A**	**G**	**R**	**V**	**Q**	**C**	**S**	**A**	**S**	**H**
Detected sequence	A					E		I				T			T			A	T		
	B							V		K		T			I			A			
	C			H								T		K				A			Y
	D					E		I		K		T						A	T		
	E		D					V		K		T						A			
	F							V		K		T	R					A	T		
	G			H						·		T	·			R		A		T	
	H			H	R		R	F				T	·	·	·	·	·	A	·	·	·
Macaque	028GCE	1 (F)						V		K		T	R		I			A	T		
		2 (B)						V		K		T			I			A			
	979BAC	1 (H)			R		R	F				T						A			
		2		H	R			F	K		T	T					Y	A	T		
	049JI	1 (H)		H	R		R	F				T						A			
		2		H	R		R	F				T	E	K				T			
	357GH	1 (G)		H								T				R		A		T	
		2 (G)		H								T				R		A		T	
	980BAC	1 (H)		H	R		R	F				T						A			
		2 (H)		H	R		R	F				T						A			

Sequences are shown alongside the SFV *M. fascicularis* reference sequence, SFVmfa (Accession number AY686197) and the sequences identified in the colony (A-H) at the earliest time point. *, where appropriate, if the sequence is present elsewhere in the colony it is indicated in parentheses. The period between time points 1 and 2 is 67 months.

The consensus sequences isolated from 980BAC and 357GH remained the same for each animal at each sampling point, though different from each other. By contrast, bulk sequencing revealed possible evolution of virus in the remaining three of the five long-term infected macaques with both synonymous and non-synonymous changes identified resulting in some amino acid substitutions, suggesting that in some hosts there may be immune pressure on SFV, though super-infection of previously undetected variants cannot be excluded with this approach. Animal 028GCE had the fewest amino acid changes: R829G and T866A were identified and restored the sequence at these positions to that of the reference strain. In 979BAC there were five amino acid differences: R797K, which reflected the reference strain sequence at this residue; E801K, A813T and C857Y, none of which were observed in any other detected variant; and A866T. In 049JI there were five amino acid differences, N788H, K790R and R847K as well as G829E and A865T (which were not observed in the other variants).

## Discussion

We have reported previously the creation of colonies of cynomolgus macaques free of STLV-I and SRV-2 [27]. This cohort of young macaques provided an opportunity to study SFV infection, over time, in isolation from other significant retroviral infections, following young animals as they matured into a breeding population. We sought to identify, by bulk sequence analysis, the likely transmission route of virus in our colony and we revealed horizontal transmission as the more likely route, consistent with reports of SFV transmission in captive primates. There was a positive correlation between incidence of SFV infection, as measured by molecular and serological approaches, and increasing age. Moreover, we have shown that the number of different tissues harbouring provirus increases with animal age consistent with findings from a study of African green monkeys [26], revealing potential reservoirs of viral sequences, but that the actual individual tissue proviral burden is independent of age. Host immunogenetic diversity may in part determine the ability of some animals to maintain their viral burden to a low level while others are unable to control their viraemia, however this was not examined in this study.

The initial study of a group of 25 macaques, ranging in age from eight months to 27.5 years, revealed an increase in SFV prevalence with macaque age, from approximately 50% across the juvenile/infant groups to 100% in macaques older than eight years. This increase in prevalence over time is consistent with data from studies of both captive and free-ranging NHP populations [10]–[12].

We looked at the prevalence of SFV in three of the infant colonies and found that a high percentage (between 59% and 71%) of founder animals were free of SFV, thus providing an opportunity to study potential infections and assess likely transmission routes in our colonies. Over the 45 months following colony establishment, detectable SFV was identified in previously negative macaques in each colony, with SFV prevalence increasing over time. SFV detectable in the periphery appeared to occur when the animals were three to four years old, at which age male cynomolgus macaques are approaching sexual maturity. Conflicts coincident with sexual maturity increase the likelihood of being scratched and bitten and hence of SFV transmission, as has been observed in tonkean macaques [10]. The birth of infants around this time, particularly in the CA5 and CA8 colonies, further suggests that detectable SFV can coincide with sexual maturity. Since viral sequences from the tissues of these animals were not available, the possibility that detection of some of these viruses is a result of prior undetectable levels of SFV cannot be excluded.

We propose that horizontal acquisition of SFV is the dominant route of virus transmission in our cohort of macaques. We investigated this further, through study of detectable nucleotide sequence variants in the different groups. We analysed a 464-bp *pol* fragment by bulk sequencing, since this gene is polymorphic and has been used to classify primate FV strains [9,12,13]. In total there were 20 distinct sequences. Crucially, we have access to dam-offspring pairs and we highlighted that vertical transmission was not a major transmission route in our colony, since 17 of 23 offspring born in the colonies were SFV negative at their first screening despite 21 dams being SFV positive. Of the six infants that were SFV positive on their first screen, sequence analysis revealed that four had SFV sequences that differed from those detected in their dams and the adult male macaques within their respective colonies. Furthermore, sequence identity between the two SFV positive infants with identical *pol* sequences to the dam and one of the potential sires is not conclusive evidence of vertical transmission since five or six other macaques within the same colonies had identical SFV sequences. Since a bulk sequencing approach was undertaken, the possibility of infection, or superinfection, with minor sequence variants cannot be excluded.

We took the opportunity to study virus sequences within individual macaques to determine the stability of the dominant virus sequence within the host over time. We compared the *pol* consensus sequences from the first and last time points (spanning a period of 67 months) in five macaques from group CA5, which had tested positive for SFV throughout the study. The dominant sequences in two of the macaques were identical across the 67-month period, whilst in macaques 49JI, 979BAC and 028GCE a combination of both synonymous and non-synonymous amino acid changes were observed, when compared with the reference SFV sequence and the sequences present in group CA5. Calattini and colleagues noted viral genetic variation within a host over a similar timeframe (six years and five months) in Tonkean macaques. However, this variation did not result in any changes in the amino acid sequence [10]. While our data suggest that in some hosts there may be immune pressure on SFV, we cannot exclude superinfection with other minor variants circulating in the colony, since we have not performed extensive deep sequencing or clonal analysis of the sequences to analyse minor species. However, the sequences identified in 49JI, 979BAC and 028GCE may represent major immune escape variants transmitting between animals. It is not possible from this study to comment on the replicative capacity of these viruses, however, since it is possible that some sequences are more stable than others. Sequence A is present at a frequency of 43% in the colony and Sequence G for example, remained unaltered in the region of *pol* studied in one of the animals followed over five and a half years. In one instance it would appear that Sequence F mutated to Sequence B, which was present at 21% in the initial population. In this case, it is not possible to say with certainty whether the major sequence has arisen through natural mutation or exposure to superinfection with a variant.

Tissue reservoirs can impact on the effective management of a specific pathogen-free colony since tissue-associated virus could be available to enter the blood circulation; we have previously reported the detection of SRV-2 in macaque tissues, whilst none was detected in the blood of these animals [28], leading to the opportunity for infections derived from animals previously considered free of virus. Since foamy viruses can infect a wide range of tissues, we were interested to determine how soon after exposure tissue infection may have occurred and the extent of infection. Tissues were available from two young SFV-infected macaques, M955C (six months old) and 956ED (two years old). The youngest macaque had SFV provirus in the MLN and SGLN only. At two years of age 956ED already had seven tissues infected with SFV including MLN and SGLN. With the exception of 176ABA, older macaques had SFV in at least six of the 11 tissue types tested and two of the macaques had SFV infection in all 11 tissues. It has been suggested that SFV persists in leukocytes, which may aid dissemination of the virus in the host [25]. This would support the view that the lymph nodes are amongst the earliest tissues infected with SFV resulting from the trafficking of infected leukocytes. Since the liver and spleen play a major role in the host response to pathogens, the presence of viral sequences in these organs from an early point may not be surprising. There were few young animals from which tissues were available, thus we remain cautious about drawing strong conclusions regarding the infection of infants, however these data tentatively highlight that animals can become infected at a very young age and that within two years of infection, SFV can be distributed widely in host tissues. It is unlikely that the proviral burden in each tissue is derived solely from infected leukocytes in the tissue, since sequences were not consistently amplified from the same tissue in all animals and often the number of viral copies in the same tissue from different animals varied considerably.

In summary, despite a large number of dam-offspring pairs for study, we have demonstrated that horizontal infection is the primary mode of SFV acquisition in our captive cohort of cynomolgus macaques, and that an increase in infection is coincident with sexual maturity. The variety of *pol* sequences detected in the groups possibly arises from mutations acquired over time as shown in a sub-population of our macaques. Potential reservoirs of virus were identified by revealing a broad biodistribution of SFV following infection; a wide range of tissue types harbour provirus by two years of age. These data on the ease of inter-animal SFV acquisition in captivity, the rapid dissemination of provirus through many tissues and the often high tissue-associated proviral loads, highlight the challenge to prevent establishment of SFV infection, by contrast with other retroviruses, in captive macaques.

## Methods

### Study groups (SG)

Purpose-bred cynomolgus macaques (*M*. *fascicularis*), housed and maintained in accordance with United Kingdom Home Office guidelines for the care and maintenance of non-human primates, were studied. Whole blood was collected from macaques into EDTA. Tissue samples (peripheral lymph node, lung, thymus, liver, spleen, small intestine, large intestine, mesenteric lymph node, kidney, tongue, salivary gland, salivary gland-associated lymph node) were collected at termination and frozen without further manipulation. DNA was extracted from whole blood or homogenised tissues using standard methods. Extraction controls (no biological material) were prepared in parallel.

SG1 comprised 25 cynomolgus macaques (14 males, 11 females). Macaques were stratified into groups according to age: Infant (0–1 year old); juvenile (1–3 years old), sub-adult (3–6 years old), young adult (6–10 years old), mid adult (10–17 years old), old adult (>17 years old). Archived tissue and DNA samples from these macaques were available from previous studies.

SG2 comprised a sub-set of six macaques from SG1 and 12 macaques from an unrelated study.

SG3 comprised three groups of macaques: CA5, CA7 and CA8. The CA5 group comprised juvenile/sub-adult macaques (aged two to three years old at time of group formation) and the CA7 and CA8 groups comprised infant macaques (nine months to one year old at time of colony formation). These groups have been previously described [27] and archived DNA and plasma samples were available from five time points over a period of four years. The initial samples from offspring were available by approximately 12 months of age. Animal moves were occasionally required in line with husbandry priorities.

### ELISA detection of virus-specific antibodies

Plasma was separated from whole blood by centrifugation at 1100 *g* for 10 min. A published SFV ELISA protocol [11] was adapted for use. Six 96-well flat-bottomed plates (Falcon, BD, Oxford, UK) were coated with 100 μl SFV-1 Gag_1-193_-GST antigen or GST control antigen diluted to 175 μg/μl in carbonate-bicarbonate buffer (10 mM Na_2_CO_3_, 40 mM NaHCO_3_; pH 9.6) and incubated at 4°C overnight. The plates were treated with 200 μl of blocking buffer (5% porcine serum (v/v), 0.05% (v/v) Tween 20 in PBS) and incubated for 1 h at room temperature. Following a wash with 200 μl per well PBST (PBS containing 0.05% (v/v) Tween 20), triplicate plasma samples were titrated in two-fold dilutions in blocking buffer from an initial 1:100 dilution and allowed to bind for 1 h at room temperature. A negative control plasma sample was included on each plate. Positive controls were plasma samples obtained from macaques previously shown to have a strong anti-SFV antibody response. The plates were washed five times as above and 50 μl of anti-human IgG conjugated to HRP diluted 1:2000 was added to the wells and the plates incubated for a further hour at room temperature. The plates were washed five times as above and 100 μl of TMB peroxidise EIA was added to each well and the plates incubated for 8 min at room temperature. The reaction was stopped by the addition of 50 μl of 2N H_2_SO_4_. The absorbance was measured at OD_450_. All ELISA-positive samples had an OD_450_ value 0.250 at a dilution of 1:100. All ELISA-negative samples had an OD_450_ value ≤0.09 at a dilution of 1:100.

### PCR amplification

A 310-bp SFV LTR fragment was amplified from 200 ng DNA in a 25 μl reaction comprising: 10 mM Tris–HCl, pH 8.3; 50 mM KCl; 2 mM MgCl_2_; 0.25 μM of each oligonucleotide (786N (5′ CAC TAC TCG CTG CGT CGA GAG TGT 3′) and 1115C (5' GGA ATT TTG TAT ATT GAT TAT CC 3')); 0.2 mM each of dATP, dCTP, dGTP and dTTP and 1.25 U AmpliTaq Gold DNA polymerase (Applied Biosystems, CA, USA). Amplification consisted of a single cycle at 94°C for 10 min, followed by 40 cycles comprising 95°C for 30 sec, 44°C for 30 sec, 72°C for 1 min and a single 10 min incubation at 72°C.

A 464-bp SFV *pol* fragment was amplified from DNA using a nested-PCR protocol. DNA (200 ng) was amplified in a 25 μl reaction comprising: 10 mM Tris–HCl, pH 8.3; 50 mM KCl; 2 mM MgCl_2_; 0.25 μM of each oligonucleotide (5951N (5' GCC ACC CAA GGR AGT TAT GTG 3') and 6500C (5' TGC KCC RTG YTC AGA GTG 3')); 0.2 mM each of dATP, dCTP, dGTP and dTTP and 2 U FastStart Taq DNA polymerase (Roche Diagnostics GmbH, Germany). First round amplification consisted of a single cycle at 94°C for 10 min, followed by 35 cycles comprising 94°C for 1 min, 38°C for 1 min, 72°C for 2 min and a single 10 min incubation at 72°C. Nested amplification was performed in an identical reaction mix using oligonucleotides 5993N (5' CCT GGA TGC AGA GYT GGA TC 3') and 6438C (5' GAY GGA GCC TTW GTG GGY TA 3') and 1 μl of the first-round product as template. Reaction conditions consisted of a single cycle at 94°C for 10 min followed by 25 cycles comprising 94°C for 1 min, 55°C for 1 min, 72°C for 2 min and a single 10 min incubation at 72°C.

To quantify the number of SFV copies, a region of SFV-1 *pol* (nt 5993–6419, GenBank accession number X54482) was cloned into pGEM®-T Easy vector (Promega UK Ltd, Southampton, Hampshire) creating plasmid pGEM-SFV1. A 10-fold dilution series was used ranging from 1 × 10^6^ copies/μl to 1 copy/μl as a PCR positive control for amplification using a previously reported method [29].

Differences in viral distribution and proviral loads were analysed between infection groups using the non-parametric Mann–Whitney test. All tests were performed using Prism v.4 (GraphPad Software; http://www.graphpad.com).

### Sequence analysis

SFV *pol* PCR products were sequenced in both directions using the BigDye Terminator v3.1 cycle sequencing kit (Applied Biosystems) and an ABI Prism 3130xl Genetic analyser (Applied Biosystems). The fragments were assembled using Ridom TraceEdit. Consensus sequence alignment and phylogenetic analyses were performed using Mega 5.0 [30]. SFV reference sequences were available from GenBank from rhesus macaque (SFVmac, accession number X83292; SFV1, X54482), cynomolgus macaque (SFVmfa, AY686197), Tonkean macaques (SFVMtoT7, DQ354076; SFVMtoTM3, DQ354079; SFVMtoTQ3, DQ354073), chimpanzee (SFVcpz, U04327) and African green monkey (SFVagm, X8329; SFV3, M74895). Nucleotide-based phylogeny was derived by both the Neighbor-Joining method using the Kimura-2-parameter substitution model and Maximum Likelihood analysis. Consistent models were obtained using each approach; data using the NJ method are shown. Evolutionary distance was analysed by the Pairwise distance calculation using the Kimura-2-parameter.

## Competing interests

The authors declare that they have no competing interests.

## Authors’ contributions

SH participated in the design of the study; performed the molecular assays, immunoassays and sequence analyses and helped to draft the manuscript; JLM participated in the design of the study; carried out the tissue viral load quantification; performed the statistical analyses and helped to draft the manuscript; MS contributed to the molecular assays; NMA and KLC participated in the design of the study and helped to draft the manuscript; NJR conceived of, and coordinated the design of, the study and drafted the manuscript. All authors read and approved the final manuscript.
